# Evaluating the Impact of Meningococcal Vaccines With Synthetic Controls

**DOI:** 10.1093/aje/kwab266

**Published:** 2021-11-09

**Authors:** Ottavia Prunas, Daniel M Weinberger, Duccio Medini, Michele Tizzoni, Lorenzo Argante

**Keywords:** effectiveness, interrupted time series analysis, invasive meningococcal disease, meningococcal infections, synthetic controls, vaccine impact, vaccines

## Abstract

Invasive meningococcal disease (IMD) has a low and unpredictable incidence, presenting challenges for real-world evaluations of meningococcal vaccines. Traditionally, meningococcal vaccine impact is evaluated by predicting counterfactuals from pre-immunization IMD incidences, possibly controlling for IMD in unvaccinated age groups, but the selection of controls can influence results. We retrospectively applied a synthetic control (SC) method, previously used for pneumococcal disease, to data from 2 programs for immunization of infants against serogroups B and C IMD in England and Brazil. Time series of infectious/noninfectious diseases in infants and IMD cases in older unvaccinated age groups were used as candidate controls, automatically combined in a SC through Bayesian variable selection. SC closely predicted IMD in absence of vaccination, adjusting for nontrivial changes in IMD incidence. Vaccine impact estimates were in line with previous assessments. IMD cases in unvaccinated age groups were the most frequent SC-selected controls. Similar results were obtained when excluding IMD from control sets and using other diseases only, particularly respiratory diseases and measles. Using non-IMD controls may be important where there are herd immunity effects. SC is a robust and flexible method that addresses uncertainty introduced when equally plausible controls exhibit different post-immunization behaviors, allowing objective comparisons of IMD programs between countries.

## Abbreviations

CrI: credible interval
CITS: controlled interrupted time series
ICD-10: *International Classification of Diseases*
IMD: invasive meningococcal disease
ITS: interrupted time series
MenB: meningococcal serogroup B
MenC: meningococcal serogroup C
RSV: respiratory syncytial virus
SC: synthetic control

*Neisseria meningitidis* (meningococcus) is a major cause of invasive bacterial disease globally, with high rates of morbidity and mortality (1–3). The incidence of invasive meningococcal disease (IMD) is low, 0.01–3.6 cases per 100,000 persons globally, but IMD is fatal in 10–15% of cases even if treated with antibiotics, and up to 20% of survivors suffer severe sequelae (1, 4). The incidence of IMD is strongly associated with age, being highest in infancy, with a second peak in adolescence and relatively high rates in older adults (1, 5). Its incidence tends to fluctuate over time and is influenced by geographical location and an interplay of various factors, such as bacterial transmissibility and virulence, immune system maturity, degree of mucosal and systemic immunity, and social habits like smoking (6–8). This complicates epidemiologic measures of the disease, even with sophisticated mathematical models (9–15).

Meningococci are classified by their capsular serogroup, and IMD is caused almost exclusively by serogroups A, B, C, W, X, and Y (6, 16, 17). Vaccination against IMD is the only effective prevention measure (1, 6, 17), and polysaccharide-protein conjugate vaccines against serogroups A, C, W, and Y are effective in eliciting both direct and indirect immunity when implemented in mass immunization programs (18–20). The first vaccine broadly protective against meningococcal serogroup B (MenB), the 4-component meningococcal serogroup B vaccine (4CMenB) (21, 22), was introduced in 2015 in the United Kingdom’s national immunization program, and high impact was demonstrated from real-world data (23).

Measuring the impact of a mass immunization program against IMD is of primary importance for public health. Observational studies can be used to monitor real-world vaccine-attributable changes in disease incidence (24). However, due to the low incidence of IMD, several years of historical data (similar in definition and recording) can be required to obtain precise effect estimates, in a period of time where natural fluctuations in IMD incidence unrelated to vaccination are likely to occur. This inevitably introduces a risk of biased estimates and misinterpretation about causal effects (25).

A variety of quasi-experimental approaches have been developed for evaluating the impact of interventions from real-world time series data. The interrupted time series (ITS) method allows for adjustments for underlying trends (26). With controlled ITS (CITS) models, external controls can be added to adjust for time-varying confounders, which could affect the outcome (27, 28). Ideally, controls are time series that are similar to the target disease but not influenced by the intervention, typically time series of the same disease in different geographical locations. However, this approach is problematic for IMD, since its epidemiology can substantially vary between countries (1–6, 17, 29, 30). A reasonable option is to use IMD cases from the same country, but in different age groups, as controls (23, 31, 32). However, there is no general gold standard for determining the most appropriate controls. Controls that similarly fit pre-immunization data may then exhibit different postimmunization behavior, and an arbitrary selection could influence the results and underestimate uncertainty in predictions.

A possible solution is to use approaches that synthesize estimates of vaccine impact that arise when using different control diseases. This can be done by fitting models with different sets of covariates and averaging the results or by using Bayesian variable selection approaches (33–36). An extension of CITS methods is the synthetic control (SC) method (33–35), in which controls are selected from a large pool of candidate time series and are weighted to build a composite control. The set of controls with best similarity to the target time series during the pre-intervention period receives more weight. SC approaches have been applied in different fields, from marketing to internet technologies (33–35). There are variations, including SC methods based on Bayesian variable selection (35), which have been used to quantify public health interventions against pneumococcal disease and pertussis (36–39).

We investigated the validity of the SC method based on Bayesian variable selection for assessing the impact of meningococcal vaccines by applying it to 2 different immunization programs, against MenB and meningococcal serogroup C (MenC) disease in England and Brazil (23, 32), respectively, using several infectious and noninfectious diseases as controls. We compared the SC performance with traditional ITS methods, with or without control time series, in scenarios where the immunization program was expected to have no effect. We then compared vaccine impact estimates of the SC method with the corresponding original assessments. Finally, we investigated which control diseases had the highest probability to be selected as predictors of meningococcal disease in infants.

## METHODS

### Data

We collected laboratory-confirmed MenC invasive disease cases, grouped by subject age and month of disease onset (January 2007 to December 2013) (31) from Brazilian public databases (40). Data from the city of Salvador were excluded because a mass vaccination campaign was implemented in 2010 following an outbreak of MenC disease (31). The MenC vaccine-eligible age groups were <1 and 1–4 years old. For England, MenB invasive disease cases were retrieved from the Public Health England national surveillance system website (41). We collected quarterly cases from the last quarter of 2011 to the first quarter of 2019; before this period, data were not stratified by age group. The MenB vaccine-eligible age groups were aged 18 to 51 weeks and 1 year (Web Appendix 1, available at https://doi.org/10.1093/aje/kwab266). Details on the 2 early-childhood immunization programs are provided in Table 1.

**Table 1:** Details on the Meningococcal Serogroup C and Meningococcal Serogroup B Vaccination Programs Implemented in Brazil and England

**Country**	**Target Time Series**	**Target Age Groups**	**Time Range**	**Vaccine Schedule**	**Start of Immunization Program**	**Evaluation Period**	**First Author, Year (Reference No)**
Brazil^a^	Monthly MenC cases	<1 year and 1–4 years	January 2007 to December 2013	3 doses at ages 3, 5, and 12–15 months	November 2010	December 2011 to December 2013	SINAN, 2016 (40)
England	Quarterly MenB cases	18–51 weeks and 1 year^b^	Fourth quarter 2011 to first quarter 2019	3 doses at ages 2, 4, and 12 months	September 2015	Fourth quarter 2016 to first quarter 2019	PHE, 2014 (41)

Abbreviations: MenB, meningococcal serogroup B; MenC, meningococcal serogroup C; PHE, Public Health England; SINAN, Sistema de Informação de Agravos de Notificação.

^a^ Cases from the city of Salvador are excluded from the analysis.

^b^ After data augmentation, as described in the Web Appendix 1.

As candidates to compose SC, we used time series of cases from several infectious/noninfectious diseases from the same country and same target age groups. We included only diseases that were unaffected by meningococcal vaccination and for which no other interventions were introduced during the considered time. In addition, we used time series of the same target disease (MenB for England, MenC for Brazil) in older age groups not eligible for the immunization program. Full lists of control time series used for Brazil and England are provided in Web Tables 1–2 and Web Appendix 2.

For the CITS, we used MenB/MenC cases in non-vaccine-eligible age groups as control time series, as done in previous analyses of the same data from Brazil and England (23, 32).

### Models to assess the impact of the vaccines

We followed the SC approach based on Bayesian variable selection (35). SC models were fitted to prevaccination targets (meningococcal time series) (36); fitted SC models were then used to generate counterfactual predictions for postvaccination periods (36, 42). The SC models relied on Bayesian variable selection to select the optimal set of candidate controls and combine them into a composite (36, 42). The specific approach used implements a Poisson model with an observation-level random intercept developed to fit over dispersed count data, available as an R (R Foundation for Statistical Computing, Vienna, Austria) package (43), as follows.

IMD cases ${y}_t$ at time *t* are modeled as a Poisson process, ${y}_t$∼ Poisson(${\lambda}_t$), with mean ${\lambda}_t$ (42, 43):

(1) \begin{align*}\log ({\lambda}_t)&={b}_0+\sum \limits_k{c}_k\ast I[ mont{h}_k=m(t)]\nonumber\\&\quad+\sum \limits_{k=1}^p{\beta}_k({\delta}_k)\ast {x}_{kt}+{b}_{c(t)}\end{align*}

where *t* = 1,2,…, is the total number of time points; ${x}_{kt}$ represents the number of cases of control disease *k* at time *t*; ${m}_t$ is a function that maps a time point to the corresponding calendar month; ${c}_k$ represents the month *k* regression coefficient; *I*[*.*] represents the indicator function; ${b}_0$ is an intercept; *p* is the total number of control diseases included in the analysis; ${\beta}_k$ (${\delta}_k$) is the regression coefficient for control disease *k*, which is given a spike-and-slab prior distribution (depending on ${\delta}_k$) in order to allow for data-driven variable selection; ${\delta}_k$ are binary random variables that are equal to 1 if the control disease *k* is included in the model or equal to 0 if it is excluded; and ${b}_{c(t)}$ is an observation specific random intercept. All the control time series were log-transformed and standardized prior to being included in the model.

The Bayesian variable selection procedure (35, 36, 42) with a spike-and-slab prior distribution was used to select variables among the candidate control time series. Each control is associated with an indicator variable, which is 1 if the control is included in the model and 0 otherwise. Specifically, we used the function “poissonBvs” (44) in R (R Foundation for Statistical Computing), which uses a Markov chain Monte Carlo sampling scheme for Bayesian inference (45). Among the values returned from the “poissonBvs” function, there is the posterior probability that the indicator variable ${\delta}_k$ is equal to 1 (“pdeltaBeta” in the package). The probability of inclusion for each control variable is computed as the proportion of Markov chain Monte Carlo iterations that include the variable in the model. We collected 10,000 posterior samples after a burn-in period of 5,000 iterations.

Since subjects in age groups not eligible for the meningococcal immunization program may have been indirectly protected by vaccination (herd immunity effects), fitting the SC models was repeated excluding IMD time series from the control sets, as a sensitivity analysis. We called ”SC1” the models that used all the controls (including those that may be affected by herd immunity), while the second implementations without meningococcal controls were called ”SC2” models.

The SC1 and SC2 models were first tested on target age groups not included in the vaccination programs, specifically on time series of IMD cases in age groups aged 5–9, 10–14, and 15–19 years (when used as a target, the respective time series was removed from the set of controls for the SC1 model). In these scenarios, if the models provide good predictions, there will be no measurable vaccine effect in any of the age groups in Brazil or England. Consequently, the counterfactuals would be close to the observed points (no indirect protection was assumed, as reported in previous analyses (23, 32)).

As a comparison, we also tested more traditional models: ITS, where no external controls are used, and CITS, where control time series are selected by the investigator and included as covariates. We tested models that included control time series for all age groups and also tested models with single age groups at a time to give an indication of whether the choice of controls influences the estimates. Two variants of ITS and CITS were implemented: One included changes in both level and slope (ITS-S and CITS-S), and the other incorporated changes in level only (ITS-L and CITS-L). Further details on the ITS and CITS models are available in the Web Appendices 3 and 4.

### Evaluation of vaccine impact

Vaccine impact was computed by comparing the total number of observed cases (*Y_obs_*) and the number of predicted counterfactual cases (*Y_cf_*) during the evaluation period *T_eval_*:

$$ \begin{align*}\mathrm{Vaccine}\ \mathrm{Impact}=1\ \hbox{--}\ \mathrm{incidence}\ \mathrm{rate}\ \mathrm{ratio},\end{align*}$$

where the incidence rate ratio equals (*Y_obs_* / *T_eval_*) / (*Y_cf_* / *T_eval_*) (36).

We excluded the first year after vaccine introduction from the evaluation period (see Table 1), to avoid evaluating the impact while vaccine uptake was not yet stable, in agreement with previous impact studies (23, 32).

## RESULTS

### The SC model accurately predicted observed meningococcal cases in the absence of vaccination

**Figure 1:**
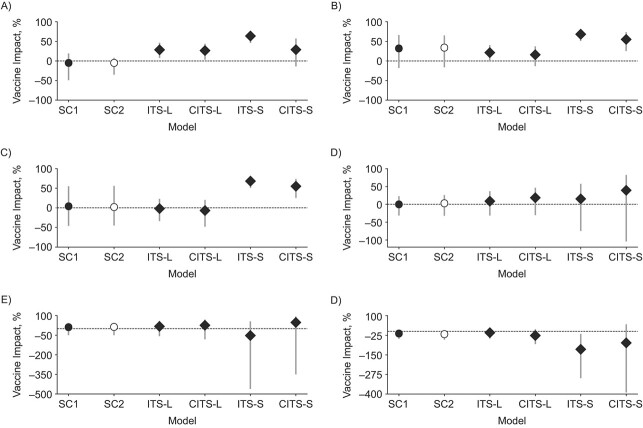
Vaccine impact estimates in non-vaccine-eligible age groups: 5–9 years (A and D), 10–14 years (B and E), and 15–19 years(C and F) in Brazil (A, B, and C), 2007–2013, and England (panels D, E, and F), 2011–2019, using different models (synthetic control 1 and 2 (SC1 and SC2) shown as circles; interrupted time series (ITS) and controlled ITS (CITS) as diamonds). CITS-L, CITS with all meningococcal serogroup B (MenB) (England)/meningococcal serogroup C (MenC) (Brazil) cases in non-vaccine-eligible age groups used as controls (excluding the target) and incorporating changes in level only; CITS-S, same as CITS-L, but incorporating changes in both level and slope; ITS-L, interrupted time series incorporating changes in level only; ITS-S, ITS incorporating changes in both level and slope; SC1, synthetic control method using all the controls available; SC2, synthetic control method excluding IMD cases in non-vaccine-eligible from the set of candidate controls.

**Figure 2:**
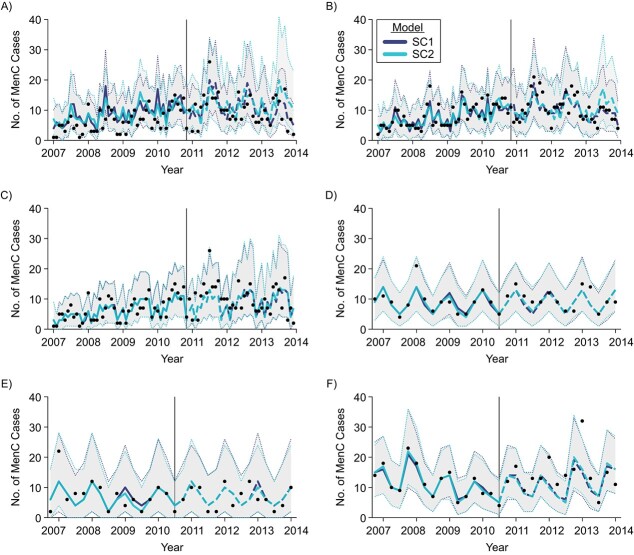
Meningococcal cases predicted by the synthetic control 1 and 2 (SC1 and SC2) models for meningococcal serogroup C (MenC) (Brazil, 2007–2013) (A, B, and C) and meningococcal serogroup B (MenB) (England, 2011–2019) (D, E, and F) disease among nonvaccinated persons in the age groups 5–9(A and D), 10–14 (B and E), and 15–19 (C and F) years. In dark blue, cases predicted with the SC method using all the controls available (SC1) (curve: best estimate; shaded region: 95% credible interval (CrI)). In light blue, cases predicted excluding MenB/MenC cases in unvaccinated age groups (SC2) (curve: best estimate; shaded region: 95% CrI). Observed data reported as black dots. The model was fitted on prevaccination data only (best fits shown as solid lines). Postintervention predictions (i.e., counterfactuals) shown as dashed lines.

Using the SC approach with meningococcal cases in nonvaccinated age groups as a target, we found no significant vaccine impact, as expected (Figure 1), and the SC1 model correctly captured the seasonal behavior of IMD cases in all age groups (Figure 2). In addition, the SC1 model accurately reproduced long-term nonlinear trends in the incidence of IMD (i.e., the decrease in MenC cases among 5- to 9- and 10- to 14-year-olds in Brazil since 2012 (Figure 2A–B), and the increase in MenB cases among 15- to 19-year-olds reported in England during the entire immunization period, compared with negative trends reported before immunization (Figure 2F)).

We tested the robustness of the SC estimates by using only non-IMD controls (SC2 model). With exclusion of IMD cases from the set of controls (i.e., using other diseases only), predictions did not change in England for any age group. In Brazil, small, statistically nonsignificant discrepancies were observed between the SC1 and SC2 predictions in age groups 5–9 and 10–14 years in the last 6–7 months of the evaluation period (Figure 2A–B).

When the ITS and CITS models were tested on nonvaccinated age groups, in some cases, an unexpected significant positive or negative impact was detected (Figure 1). In general, a change in the slope negatively affected predictions: ITS and CITS predictions both improved when the change was in level only and not in slope (i.e., models ITS-L and CITS-L).

### Impact estimates in Brazil and England

**Figure 3:**
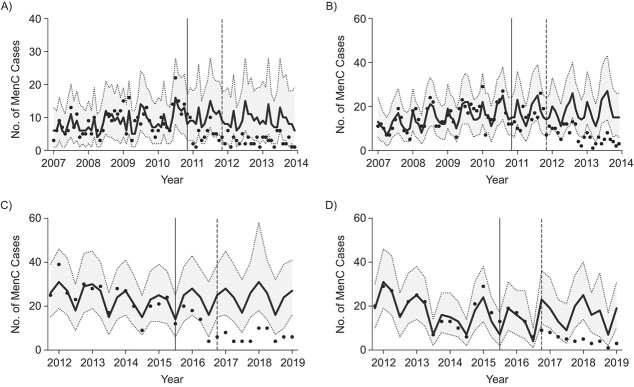
Meningococcal cases predicted by the synthetic control 1 (SC1) model for meningococcal serogroup C (MenC) disease (Brazil, 2007–2013) in vaccine-eligible age groups of <1-year-olds (A) and 1- to 4-year-olds (B) and meningococcal serogroup B (MenB) disease (England, 2011–2019) in 18- to 51-week-olds (C) and 1-year-olds (D). In black, cases predicted with the SC method (curve: best estimate; shaded region: 95% credible interval (CrI)). Observed cases are shown as black dots. Solid black vertical lines indicate the introduction of the vaccination campaign. Dashed gray vertical lines indicate the initial point for measuring impact.

When using IMD cases in vaccinated age groups as the target disease, the SC1 model fitted prevaccination data well, even in the presence of nontrivial incidence patterns, such as a trend inversion between 2014 and 2015 in England (Figure 3). Reported meningococcal disease incidences declined in both countries after the introduction of infant routine immunization programs, and the observed values declined relative to the counterfactual predictions. In Brazil, we measured a 69% (95% credible interval (CrI): 51, 80) vaccine impact on MenC cases in <1-year-old infants associated with vaccination. In children aged 1–4 years, the impact was estimated to be 64% (95% CrI: 55, 70). In England, we estimated a 75% reduction (95% CrI: 69, 80) in 18- to 51-week-old infants. In 1- and 2-year-olds, the reduction was, respectively, 72% (95% CrI: 65, 79) and 58% (95% CrI: 38, 71). These vaccine impact estimates were in agreement with previous assessments based on time series methods (23, 32) (Web Appendix 5 and Web Tables 3–4).

Vaccine impact estimates from the ITS-L and CITS-L models were in line with SC1 model results in all age groups and countries, though in 1- to 4-year-olds in Brazil, the predicted impact was slightly higher (Web Figure 1). Vaccine impact estimates were not affected by the choice of controls (Web Figure 2).

Conversely, including a linear trend component (change in slope) led to high variability among vaccine impact estimates in all age groups and both countries depending on which controls were included, if any (Web Figures 1–3). In the <1-year-olds age group in Brazil, a simple ITS model without covariates estimated an 81% decline in incidence while a model with all covariates estimated a 75% decline, and results from CITS models with a single covariate ranged between them (Web Figure 3). Similarly, in 18- to 51-week-olds in England, we found impact values ranging from 48% for ITS to 62% for the CITS model with all covariates (Web Figure 3).

### IMD cases in older unvaccinated age groups are consistently among the best predictors of infant IMD caused by the same serogroup

Even when the most frequently selected time series varied by age group and country, there was a common general pattern, since the IMD (MenB/MenC) time series in noneligible age groups was consistently among the 3 predictors with highest probability of inclusion.

**Figure 4:**
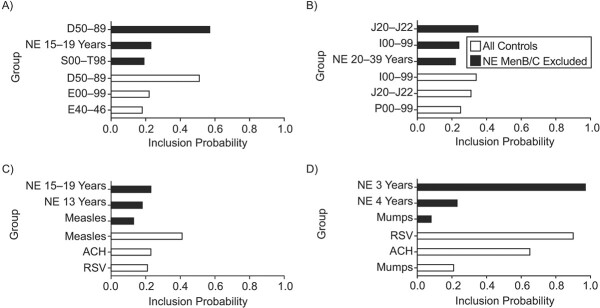
Top 3 selected controls with highest probability of inclusion, for the <1-year-olds age group and 1- to 4-year-olds age group in Brazil(A and B, respectively), 2007–2013; and for the 18- to 51-week-olds age group and 1-year-olds age group in England (C and D, respectively), 2011–2019. We report results using all the controls (black bars) and a subset where meningococcal serogroup B (MenB)/ meningococcal serogroup C (MenC) cases in non-vaccine-eligible age groups were excluded (white bars). ACH, aggregated variable with all the controls summed together; D50–89, diseases of blood and blood-forming organs and certain disorders involving the immune mechanism; E00–99, endocrine, nutritional, metabolic disorders; E40–46, malnutrition; I00–99, diseases of the circulatory system; J20–J22, bronchitis, bronchiolitis, and unspecified acute lower respiratory infection; NE, noneligible age group; P00–99, perinatal diseases; RSV, respiratory syncytial virus; S00–T98, injury, poisoning, and consequences of external causes.

For Brazil, a larger number of candidate controls was available (see full list of 36 controls in Web Table 1) than for England (Web Table 2). Figure 4 (“All the controls*”* panels) displays the 3 most selected time series according to probability of inclusion to fit pre-immunization MenC cases for age groups <1 and 1–4 years, which included not only MenC cases in older age groups, but also other infectious and noninfectious disease time series. The most selected predictor of MenC in <1-year-olds was “diseases of blood and disorders involving the immune mechanism” (*International Classification of Diseases*, *Tenth Revision* (ICD-10) code D50–89, probability of inclusion (Prob) = 0.57), followed by MenC in adolescents aged 15–19 years (Prob = 0.23) and “injury, poisoning, and consequences of external causes” (ICD-10 code S00-T98, Prob = 0.19). For MenC in 1- to 4-year-olds the most selected predictor of MenC was first “other acute lower respiratory infections” (ICD-10 codes J20–J22, Prob = 0.35), then “diseases of the circulatory system” (ICD-10 I00–99, Prob = 0.24) and MenC cases among 20- to 39-year-old adults (Prob = 0.22).

In England, as shown in Figure 4 (“All controls”), MenB incidence in the non-vaccine-eligible age groups was predominantly selected among the top controls. In particular, MenB cases in the 3-year-olds age group was selected with Prob = 0.97 to predict MenB in 1-year-old children. For the 18- to 51-week age group, posterior inclusion probabilities were lower than 50%. The most selected control time series was MenB cases in 15- to 19-year-olds with Prob = 0.23, similar to MenC in Brazil. Also, 2 childhood infectious diseases, measles and mumps, were selected among the best controls for the 18- to 51-week age group and 1-year-olds, respectively, but with a relatively lower probability (respectively, Prob = 0.13 and Prob = 0.08).

### SC predictions are not affected by the exclusion of IMD cases in unvaccinated age groups

The robustness of the SC approach was tested by repeating the analysis with the exclusion of IMD cases of the same serogroup in older unvaccinated age groups (the SC2 model). MenB and MenC controls could be influenced by indirect (herd immunity) effects and removing IMD controls of the same serogroup allowed us to relax our initial assumption (i.e., that indirect effects are negligible).

**Figure 5:**
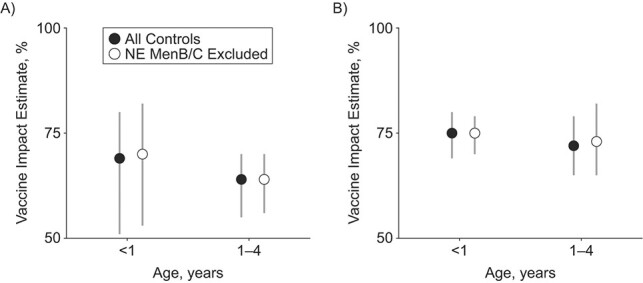
Vaccine impact estimates for meningococcal serogroup C (MenC) (Brazil, 2007–2013) disease in the vaccine-eligible age groups of <1- and 1- to 4-year-olds (A) and meningococcal serogroup B (MenB) (England, 2011–2019) disease in the 18- to 51-week-olds and 1-year-olds (B) when using the synthetic control 1 and 2 (SC1 and SC2) models (black and white dots). 95% credible intervals (CrIs) are shown as gray lines. Vaccine impact estimates using all the controls available are shown as black dots. Vaccine impact estimates excluding MenB/MenC cases in unvaccinated age groups are shown as white dots. NE, noneligible age group.

For Brazil, excluding non-vaccine-eligible IMD cases from the set of controls (SC2) did not change the goodness of fit and accuracy of predictions, while for England the performance was lower (Web Figure 4 and deviation information criterion and mean absolute error metrics displayed in Web Tables 5–6 and Web Appendix 6). For both countries and age groups, impact estimates were robust, with almost coincident best estimates and overlapping 95% CrIs (Figure 5).

Looking at the controls selected with the highest probability, the SC2 model often selected other respiratory infections or airborne diseases. Specifically, for England, we found that measles and respiratory syncytial virus (RSV) were frequently selected as predictors of meningococcal disease incidence (Figure 4 “NE MenB excluded” panel). In particular, the incidence of RSV is associated with a probability of inclusion Prob = 0.90 to predict MenB in 1-year-olds. In Brazil, a specific time series for RSV was not available among the controls. However, acute lower respiratory infections (ICD-10 J20–J22) consistently appeared among the top 3 controls for 1- to 4-year-olds (Figure 4, “NE MenC excluded”). Codes J20–J22 refers to bronchitis, bronchiolitis, and other acute lower respiratory infections, including bronchitis and bronchiolitis due to RSV.

## DISCUSSION

We reanalyzed data from 2 large infant immunization campaigns in Brazil (against MenC disease) and England (against MenB disease) using a SC approach. The 2 settings differed in many aspects, including meningococcal serogroup, vaccine type, disease seasonality patterns, age and socioeconomic status of the population, and public health system. Nevertheless, findings with the SC method were in good agreement with those from the original studies (Web Tables 3/4 (23, 32)).

Control variables are crucial when predicting infectious diseases like IMD, given the large number of clinical, epidemiologic, social, and environmental factors that could influence its behavior (1). Some of these factors are probably strictly related to the pathogen studied, while it is reasonable to assume others are shared with other diseases. For this reason, we included, as candidate controls, infectious and noninfectious disease cases in infants of the same age as those vaccinated, following the approach previously applied to pneumococcal vaccines (36). We also included MenB and MenC cases in older age groups that were not eligible for the vaccine program, similar to the original analyses run in England and Brazil (23, 32). We then investigated which control diseases should be selected to compose the SC.

MenB and MenC cases in noneligible age groups were consistently among the most frequently selected controls in England and Brazil, respectively. Examination ofnon-IMD diseases associated with infant IMD identified RSV disease and measles as among the best predictors for 1-year-old and 18- to 51-week-old infants, respectively, in England. An association between IMD and RSV and measles cases has been reported in some epidemiologic studies (46–49), although others did not detect any clinical association (50, 51). One recent study found that measles could reduce humoral immune memory, thereby generating potential vulnerability to future infections (52). In Brazil, we found bronchitis/bronchiolitis and other acute respiratory infections to be good predictors of MenC cases. Both time series included RSV disease cases (36, 53).

Our test of the performance of ITS and CITS designs found that a linear trend component (change in slope) may bias the results towards seeing an effect of vaccination when no vaccine impact is expected. This was probably due to the linear trend component incorrectly projecting an increasing or decreasing trend even after adjusting for covariates. Removing the linear trend component improved ITS and CITS performances. CITS-L showed performances similar to the SC method, which is not surprising since the most frequently selected controls in the SC were the ones incorporated in CITS (i.e., IMD cases in non-vaccine-eligible agegroups).

In general, SC approaches based on Bayesian variable selection are appealing because they allow appropriate controls to be identified in situations where the choice is not obvious, and various plausible controls exist that may generate different counterfactual predictions. For example, when 2 or more plausible controls (e.g., IMD cases in different age groups) fit equally well for pre-immunization data but differ in the postimmunization period, results will be influenced by selecting one control or all of them. Instead, the SC method probabilistically generates posterior counterfactuals, whose CrIs will also include uncertainty due to diverging controls in the postimmunization period. In this way SC efficiently handles uncertainty due to control selection, enabling more reliable counterfactuals to be built. In any case, it is usually beneficial to test different models (such as ITS, CITS, and SC) as a comparison of results may reveal differences that require further investigation to address possible sources of confounding.

SC, ITS, and CITS models are all quasi-experimental approaches in which interventional effects are evaluated relative to a predicted counterfactual, and not with respect to a similar population that received a placebo. Therefore, interpretation of results concerning the causality of such interventional effects should always be done cautiously and in light of the assumptions made when generating counterfactuals. The SC method relies on 2 major assumptions: 1) the time series of candidate control diseases must be unaffected by the vaccine under study, and 2) any change in the relationship between the target disease and components of the SC over time must be caused by the vaccine (36). If the SC assumptions are fulfilled, then the difference between observed incidence and counterfactual may be interpreted as an indication of a causal effect of the vaccine (25, 36). However, no firm conclusions can be made on causality: As for other observational study designs, it is unlikely that confounding can be completely eliminated (36).

Our work has some limitations. Using time series of IMD cases in non-vaccine-eligible age groups as controls comes with inherent risks of generating biased impact estimates. Meningococcal vaccination may indirectly protect unvaccinated subjects (12, 54), so a reduced risk of IMD in nonvaccinated age groups would lead to an underestimation of vaccine impact (24, 25). In particular, it has been shown that MenC vaccines induce indirect protection, specifically when targeting larger portions of the population that also include adolescents (15, 55). However, previous studies reported no evidence of indirect effects in both of the investigated settings (23, 31, 32). We nevertheless repeated all the analyses excluding meningococcal cases in non-vaccine-eligible age groups from the set of controls. The results were robust even with this exclusion. In some circumstances, the SC model failed to identify an appropriate set of controls, such as with 18- to 51-week-old infants in England (Figure 4). Here, the SC model was still able to produce a reliable counterfactual with only the intercept and seasonal components (Figure 3C).

Present results suggest that the SC model could be successfully applied to evaluate meningococcal immunization campaigns targeting adolescents and adults, where indirect effects could hamper a correct assessment of the overall impact. SC correctly adjusted for nontrivial changes in incidence of IMD and efficiently handled model uncertainty about which controls to include through Bayesian variable selection. Also, our finding that IMD may be associated with measles and RSV disease should be further investigated to uncover possible common causal factors.

In conclusion, we showed that the SC model is a promising approach for estimating the impact of meningococcal immunization programs. Its general applicability in different contexts and its efficiency in automatically addressing uncertainty about selection of controls allows for an objective comparison between meningococcal vaccines and immunization strategies in different countries, offering a valid alternative for public health decision making.

## Supplementary Material

Web_Material_kwab266
