# Supplementary material for: Evaluating the Impact of Meningococcal Vaccines With Synthetic Controls
Source: Am J Epidemiol. 2021 Nov 9;191(4):724–34. doi: 10.1093/aje/kwab266 (PMC8971084; doi:10.1093/aje/kwab266)
Supplement: Web_Material_kwab266 [file web_material_kwab266.pdf]

Web Material

Evaluating the Impact of Meningococcal Vaccines With Synthetic Controls

Ottavia Prunas, Daniel M. Weinberger, Duccio Medini, Michele Tizzoni, and Lorenzo Argante

Table of Contents

Web Appendix 1. Details on data collection in England

Web Appendix 2. Time series used as components of the synthetic control model

Web Table 1. Time Series Used as Components of the Synthetic Control in Brazil

Web Table 2. Time Series Used as Components of the Synthetic Control in England

Web Appendix 3. Interrupted time series (ITS) and controlled ITS (CITS) models

Web Appendix 4. Performances of the synthetic control model, ITS and CITS models, tested on vaccine-eligible age groups

Web Figure 1. Vaccine Impact Estimates for MenC (Brazil) Disease in the <1 and 1–4 Years-Old (Panel A) and MenB (England) Disease in the 18–51-Weeks and 1-Year-Old (Panel B) Vaccine-Eligible Age Groups When Using the SC1 and SC2 Models (Black and White Dots) and ITS and CITS Models (Black Diamonds)

Web Figure 2. Vaccine Impact Estimates for MenC (Brazil) Disease in the <1 and 1–4 Years-Old (Panels A and B) and MenB (England) Disease in the 18–51-Weeks-old and 1-Year-Old (Panels C and D) Vaccine-Eligible Age Groups When Using ITS and CITS Without Linear Trend Component

Web Figure 3. Vaccine Impact Estimates for MenC (Brazil) Disease in the <1 and 1–4 Years-Old (Panels A and B) and MenB (England) Disease in the 18–51-Weeks-old and 1-Year-Old (Panels C and D) Vaccine-Eligible Age Groups When Using ITS and CITS With Linear Trend Component

Web Figure 4. Comparison Between Predictions Generated by the Synthetic Control Model for MenC (Brazil) Disease in the <1 and 1–4 Years-Old (Panels A and B) and MenB (England) Disease in the 18–51-Weeks-old and 1-Year-Old (Panels C and D) Vaccine-Eligible Age Groups

Web Appendix 5. Comparison of vaccine impact estimates between the synthetic control model and previously published methods

Web Table 3. Vaccine Impact Estimates in Brazil

Web Table 4. Vaccine Impact Estimates in England

Web Appendix 6. Mean absolute error and deviation information criterion metrics

Web Table 5. DIC and MAE Estimates for Each Set of Controls and Age Groups Eligible for Vaccination in Brazil

Web Table 6. DIC and MAE Estimates for Each Set of Controls and Age Groups Eligible for Vaccination in England

Web Appendix 1. Details on data collection in England

In England, serogroup B (MenB) invasive meningococcal disease (IMD) time series, by quarter of year, of subjects eligible for the national immunization program were aggregated in 2 wide age groups (<1 and 1–4 years-old). Previous vaccine impact analyses considered MenB annual cases in finer age groups (i.e., 18–51 weeks-old and 1 year-old age groups) (1). To be consistent with previous analyses, we derived quarterly MenB cases in the 18–51 weeks-old and 1 year-old age groups, assuming that the proportion of cases by age group was maintained at a quarterly level (e.g., if cases in the 18–51 weeks-old age group were 80% of the cases in the <1 year-old age group, we used the same proportion to derive time series on a quarterly basis).

Web Appendix 2. Time series used as components of the synthetic control model

The complete list of control time series used in Brazil and England, respectively, are shown in Web Table 1 and Web Table 2. Control time series in Brazil were publicly available (2), while we downloaded control time series in England from the Public Health England website (3). Besides, we collected IMD time series in the non-vaccine-eligible age groups. MenC cases are in the age groups 5–9; 10–14; 15–19; 20–39; and 40–59 years-old in Brazil (2). MenB cases are in the age groups 5–9; 10–14; 15–19; 20–24; 25–44; 45–64; and ≥65 years-old (3). All the other control diseases are in the age groups <1 and 1–4 years-old.

Web Table 1. Time Series Used as Components of the Synthetic Control in Brazil

Grouping scheme	ICD-10	Description	Exclusions
MenC		Meningococcal C cases in non vaccine-eligible age groups	
ICD-10 chapters	C00-D48	Neoplasms	A40.3, B95
	D50-89	Diseases of blood and blood-forming organs and certain disorders involving the immune mechanism	
	E00-99	Endocrine, nutritional, metabolic disorders	
	H00-99_SY	Diseases of the ear and mastoid process	H10, H65, H66
	I00-99	Diseases of the circulatory system	
	K00-99	Diseases of the digestive system	

	L00-99	Disease of the skin	
	M00-99	Diseases of the musculo-skeletal system	
	N00-99	Diseases of the genito-urinary system	
	P00-99	Perinatal diseases	
	Q00-99	Congenital malformations, deformation and chromosomal abnormalities	
	R00-99	Symptoms, signs and abnormal clinical and laboratory findings, not elsewhere classified	
	S00-T99	Injury, poisoning and consequences of external causes	
	U00-99	Codes for special purposes	
	V00-Y99	External causes	
	Z00-99	Factors influencing health status and contact w/ health workers	
Other grouped outcomes	A10_B99_nopneumo	Certain infectious and parasitic diseases, except intestinal	A40.3, B95
	B20-24	HIV	
	E10-E14	Diabetes	
	E40-E46	Malnutrition	
	I60-I64	Stroke	
	J20-J22	Bronchitis, bronchiolitis and unspecified acute lower respiratory infection	
	P05-P07	Premature delivery and low birth weight	
	ACH_NOJ	All non-respiratory hospitalizations	J00-J99, F and O chapters
Specific outcomes	A17	Tuberculosis of nervous system	
	A18	Tuberculosis of other organs	
	A19	Miliary tuberculosis	
	A41	Other septicemia	
	B34	Viral infection of unspecified site	
	B96	Other specified bacterial agents as the cause of diseases classified to other chapters	
	B97	Viral agents as the cause of diseases classified to other chapters	
	B99	Other and unspecified infectious diseases	
	K35	Appendicitis	
	K80	Cholelithiasis	
	N39	Urinary tract infection (UTI)	

HIV: human immunodeficiency virus; ICD-10: International Classification of Diseases, 10th Revision.

Web Table 2. Time Series Used as Components of the Synthetic Control in England

**Description**
Meningococcal B cases in non-vaccine-eligible age groups
Respiratory syncytial virus (RSV)
Pertussis
Measles
Mumps
Meningococcal C
Meningococcal W
Meningococcal Y

Web Appendix 3. Interrupted time series (ITS) and controlled ITS (CITS) models

In a simple ITS model, IMD cases were assumed to follow a Poisson process $y_t \sim$Poisson(λ_t), with mean λ_t (4, 5):

$$\log(\lambda_t) = b_0 + \sum_k c_k * I[month_k = m(t)] + \beta_1 * index_t + \log(offset) + \gamma_1 * spl1_t + \gamma_2 * spl2_t \quad \text{(Eq1)}$$

where t = 1,2,..., is the total number of time points; m_t is a function that maps a time point to the corresponding calendar month; c_k represents the month *k* regression coefficient; *I[.]* represents the indicator function; b_0 is an intercept; $index_t$ is a time variable with values from 1 to the total number of time points; $offset$ is a vector of ones; $spl1_t$ and $spl2_t$ are two linear splines to capture post-vaccine changes. $spl1_t$ refers to the post-vaccine period, $spl2_t$ refers to the post-evaluation period.

In the CITS model, we added control variables as covariates:

$$\log(\lambda_t) = b_0 + \sum_k c_k * I[month_k = m(t)] + \beta_1 * index_t + \sum_{k=1}^{p} \beta_k * x_{kt} + \log(offset) + \gamma_1 * spl1_t + \gamma_2 * spl2_t$$

(Eq2)

As previously:

t = 1,2,..., is the total number of time points; m_t is a function that maps a time point to the corresponding calendar month; c_k represents the month *k* regression coefficient; *I[.]* represents the indicator function; b_0 is an intercept; $index_t$ is a time variable with values from 1 to the total number of time points; β_k is the regression coefficient for control disease *k*; $offset$ is a vector of ones; $spl1_t$ and $spl2_t$ are two linear splines to capture post-vaccine changes. $spl1_t$ refers to the post-vaccine period, $spl2_t$ refers to the post-evaluation period.

For ITS/CITS, the impact is computed comparing Y_pred (i.e. predicted outcome based on the intervention and an assumed linear model) with Y_cf (counterfactual prediction):

$$\text{Vaccine Impact} = 1 - Y_{pred} / Y_{cf}$$

In the main text, we refer to ITS-L and CITS-L models (only change in level), where no linear trend is included ($\beta_1 * index_t$ is set to 0). ITS-S and CITS-S refer to models that account for both level and slope change, and they include the linear trend component.

Web Appendix 4. Performances of the synthetic control model, ITS and CITS models, tested on vaccine-eligible age groups

In the following figures, we show vaccine impact estimates produced by the synthetic control, ITS and CITS models. In the CITS model, we used as controls all IMD cases in the non-vaccine-eligible age groups. Specifically, in Brazil we used MenC cases in 5–9; 10–14; 15–19; 20–39; and 40–59 years-old age groups. In England, controls were MenB cases in 3; 4; 5–9; 10–14; 15–19; and 20–24 years-old age group.

Web Figure 1. Vaccine Impact Estimates for MenC (Brazil) Disease in the <1 and 1–4 Years-Old (Panel A) and MenB (England) Disease in the 18–51-Weeks and 1-Year-Old (Panel B) Vaccine-Eligible Age Groups When Using the SC1 and SC2 Models (Black and White Dots) and ITS and CITS Models (Black Diamonds)

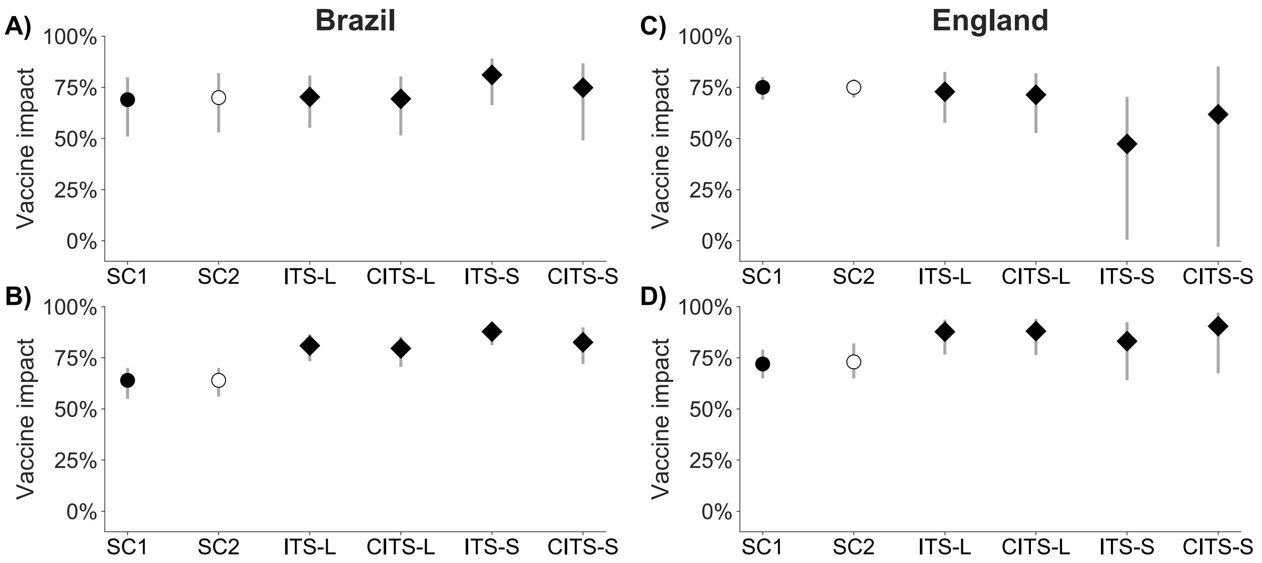

CI: credible interval; CITS: controlled ITS with all MenB (England)/MenC (Brazil) cases in non-vaccine-eligible age groups used as controls; CITS-L: CITS incorporating changes in level only; CITS-S: CITS incorporating changes in level and slope; ITS: interrupted time series; ITS-L: ITS incorporating changes in level only; ITS-S: ITS incorporating changes in level and slope; SC1: synthetic control method using all the controls available; SC2: synthetic control method excluding IMD cases in non-vaccine-eligible age groups from the controls.
95%CIs are shown as grey lines

Web Figure 2. Vaccine Impact Estimates for MenC (Brazil) Disease in the <1 and 1–4 Years-Old (Panels A and B) and MenB (England) Disease in the 18–51-Weeks-old and 1-Year-Old (Panels C and D) Vaccine-Eligible Age Groups When Using ITS and CITS Without Linear Trend Component

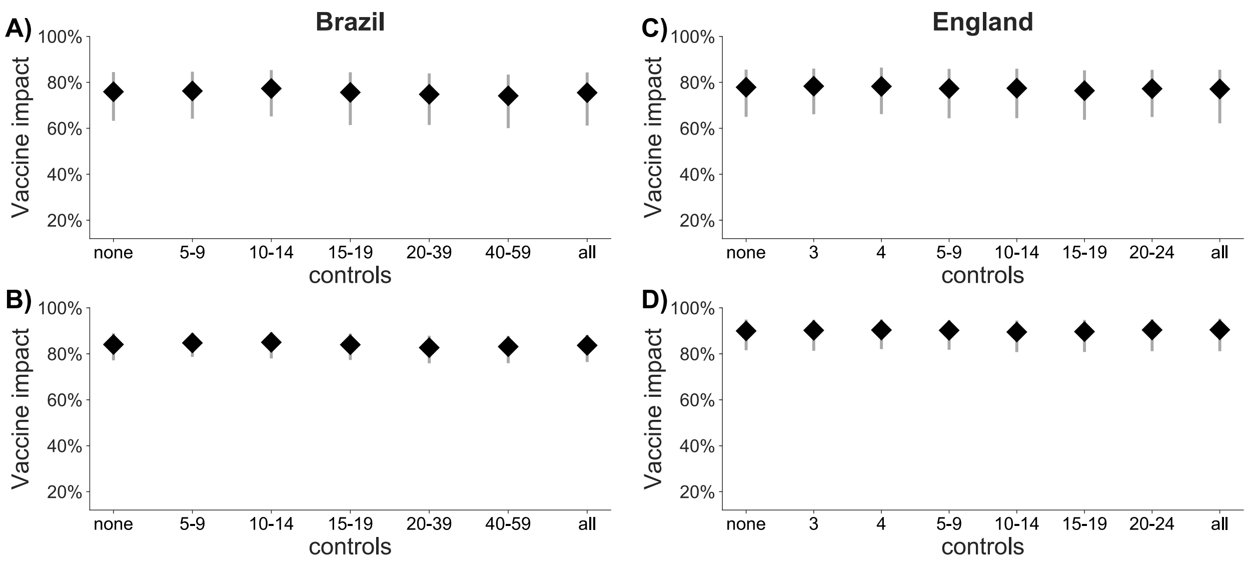

CI: credible interval; CITS: controlled ITS with MenB (England)/MenC (Brazil) cases in non-vaccine-eligible age groups used as controls; IMD: invasive meningococcal disease; ITS: interrupted time series.
95%CIs are shown as grey lines
In ITS and CITS models, no linear trend component is included (change in level only). X-axis labels: 'none' refers to a ITS without controls; 'all' refers to a CITS with all the IMD cases in non-vaccine-eligible age groups used as controls; age groups (5–9; 10–14; 15–19 years-old; etc..) in the x-axis label refer to the single control used in the CITS.

Web Figure 3. Vaccine Impact Estimates for MenC (Brazil) Disease in the <1 and 1–4 Years-Old (Panels A and B) and MenB (England) Disease in the 18–51-Weeks-old and 1-Year-Old (Panels C and D) Vaccine-Eligible Age Groups When Using ITS and CITS With Linear Trend Component

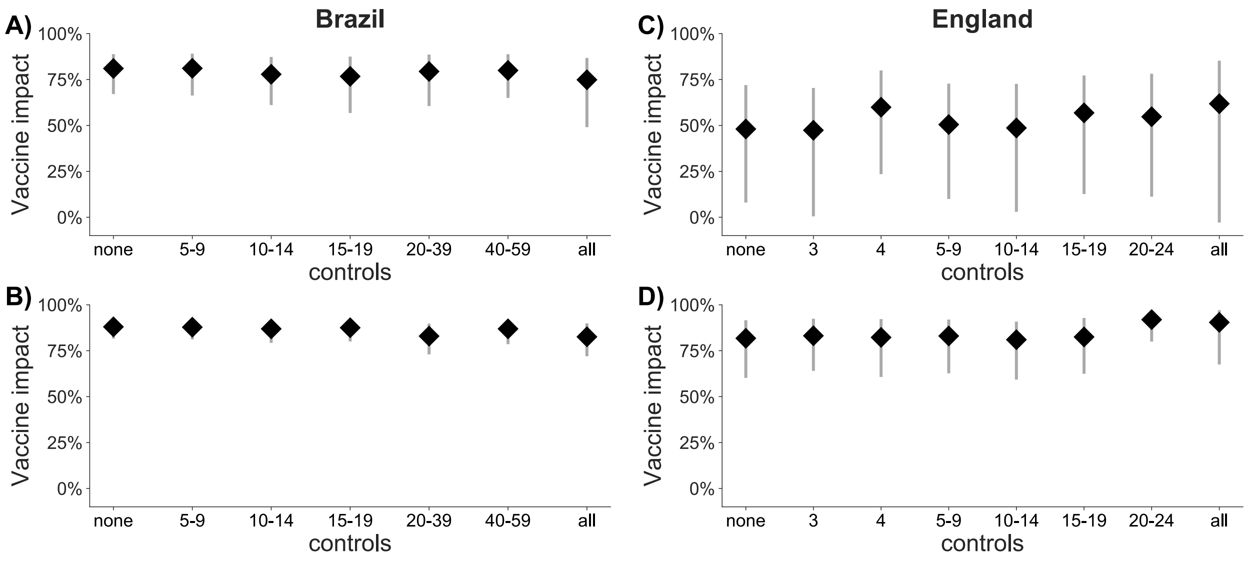

CI: credible interval; CITS: controlled ITS with MenB (England)/MenC (Brazil) cases in non-vaccine-eligible age groups used as controls; IMD: invasive meningococcal disease; ITS: interrupted time series.
95%CIs are shown as grey lines
In ITS and CITS models, a linear trend component is included (change in both level and trend). X-axis labels: 'none' refers to a ITS without controls; 'all' refers to a CITS with all the IMD cases in non-vaccine-eligible age groups used as controls; age groups (5–9; 10–14; 15–19 years-old; etc..) in the x-axis label refer to the single control used in the CITS.

Web Figure 4. Comparison Between Predictions Generated by the Synthetic Control Model for MenC (Brazil) Disease in the <1 and 1–4 Years-Old (Panels A and B) and MenB (England) Disease in the 18–51-Weeks-old and 1-Year-Old (Panels C and D) Vaccine-Eligible Age Groups

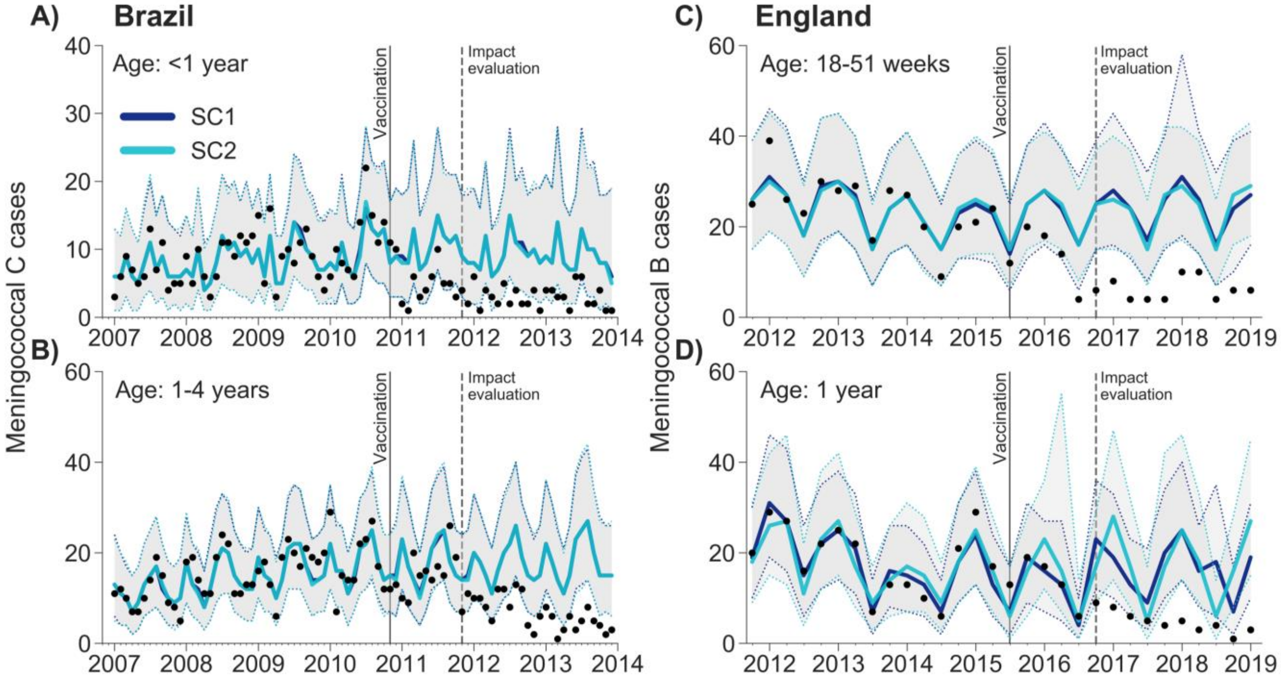

CI: credible interval; MenB: meningococcal serogroup B; MenC: meningococcal serogroup C; SC: synthetic control. In blue, cases predicted with the synthetic control method using all the controls available (SC1) (curve: best estimate; shaded region: 95%CI). In cyan, cases predicted excluding MenB/MenC cases in unvaccinated age groups (SC2) (curve: best estimate; shaded region: 95%CI). Observed cases are shown as black dots. Solid black vertical lines indicate the introduction of the vaccination campaign. Dashed grey vertical lines indicate the initial point for measuring impact.

Web Appendix 5. Comparison of vaccine impact estimates between the synthetic control model and previously published methods

Web Table 3. Vaccine Impact Estimates in Brazil

Method	Age group	Evaluation period	Impact	95%CI
SC	<1 y	Dec 2011 – Dec 2013	0.69	0.51, 0.80
GLS + AR1 (6)	<1 y	Dec 2011 – Dec 2013	0.66	0.45, 0.87
SC	1–4 y	Dec 2011 – Dec 2013	0.64	0.55, 0.70
GLS + AR1 (6)	1–4 y	Dec 2011 – Dec 2013	0.52	0.33, 0.71

AR1: first-order autoregressive; CI: confidence/credible interval; GLS: generalized least square; SC: synthetic control; y: years-old.

Web Table 4. Vaccine Impact Estimates in England

Method	Age group	Evaluation period	Impact	95%CI
SC	18–51 w	Q4 2016 – Q1 2019	0.75	0.69, 0.80
SC	1 y	Q4 2016 – Q1 2019	0.72	0.65, 0.79
SC	18 w to 1 y	Q4 2017 – Q3 2018	0.76	0.70, 0.80
Poisson regression (1)	18 w to 1 y	Depends on age group[a]	0.75	0.64, 0.81

CI: confidence/credible interval; SC: synthetic control; w: weeks-old; y: years-old.

[a]18–51 w: Sep 2016 – Aug 2018; 1 y: Sep 2017 – Aug 2018

Web Appendix 6. Mean absolute error and deviation information criterion metrics

To evaluate the goodness of fit in the pre-intervention period, we used the deviation information criterion (DIC) metric; whereas we used the mean absolute error (MAE) as a measure of prediction accuracy in the post-intervention period. The definitions of all the metrics are presented below, given that Y_i is the observed target time series at the time *i* and Y_{pred_i} is the predicted target time series at the time *i*.

- The DIC metric trades off a measure of model adequacy against a measure of complexity and is defined as: $DIC = \overline{D} + p_D$, with θ the set of parameters, $\overline{D} = E[-2logp(Y|\theta)]$ the posterior mean of the deviance and p_D the effective number of parameters.
- The MAE is a measure of the average of the absolute errors between predicted and true values and is defined as: $MAE = \frac{1}{n}\sum_{i=1}^{n}|Y_i - Y_{pred_i}|$

We computed DIC and MAE metrics to evaluate the performances of the SC1 and SC2 models on vaccine-eligible age groups.

The DIC metric was calculated over the pre-intervention period in both England and Brazil. The MAE metric was calculated in England over the first 4 points post-vaccination assuming the vaccination was not yet effective. This assumption is reasonable in 1 year-old children; however, it is no longer valid for the 18–51 weeks-old age group. In this case, the evaluation metric was computed over the last 2 points before vaccination and the first point post-vaccination. In Brazil, instead, we observed a rapid decrease in the number of cases starting from the first months after the intervention. We thus computed MAE estimate using the last 3 points before and the first point after the vaccination.

Web Table 5. DIC and MAE Estimates for Each Set of Controls and Age Groups Eligible for Vaccination in Brazil

Age group	Model	DIC	MAE
<1 y	SC1	253.92	2.5
	SC2	**253.67**	2.5
1–4 y	SC1	280.19	3.25
	SC2	**279.96**	**2.75**

DIC: deviation information criterion; IMD: invasive meningococcal disease; MAE: mean absolute error; SC1: synthetic control method using all the controls available; SC2: synthetic control method excluding IMD cases in non-vaccine-eligible age groups from the controls; y: years old.

Web Table 6. DIC and MAE Estimates for Each Set of Controls and Age Groups Eligible for Vaccination in England

Age group	Model	DIC	MAE
18–51 w	SC1	**98.27**	**3**
	SC2	98.54	3.67
1 y	SC1	88.26	**0.75**
	SC2	95.22	3

DIC: deviation information criterion; IMD: invasive meningococcal disease; MAE: mean absolute error; SC1: synthetic control method using all the controls available; SC2: synthetic control method excluding IMD cases in non-vaccine-eligible age groups from the controls; w: weeks-old: y: years old.

REFERENCES

1. Ladhani SN, Andrews N, Parikh SR, et al. Vaccination of Infants with Meningococcal Group B Vaccine (4CMenB) in England. N Engl J Med. 2020;382(4):309-317.

2. Sistema de Informação de Agravos de Notificação (SINAN). Meningitis [In Portugese]: Portal do Governo Brasileiro. http://portalsinan.saude.gov.br/meningite. Published March 8, 2016. Updated April 16, 2019. Accessed May 26, 2020.

3. Public Health England. Meningococcal disease: guidance, data and analysis: UK Government. https://www.gov.uk/government/collections/meningococcal-disease-guidance-data-and-analysis. Published July 31, 2014. Updated December 20, 2019. Accessed May 26, 2020.

4. Shioda K, Schuck-Paim C, Taylor RJ, et al. Challenges in Estimating the Impact of Vaccination with Sparse Data. Epidemiology. 2019;30(1):61-68.

5. Bruhn CA, Hetterich S, Schuck-Paim C, et al. Estimating the population-level impact of vaccines using synthetic controls. Proc Natl Acad Sci U S A. 2017;114(7):1524-1529.

6. de Moraes C, de Moraes JC, da Silva GD, et al. Evaluation of the impact of serogroup C meningococcal disease vaccination program in Brazil and its regions: a population-based study, 2001-2013. Mem Inst Oswaldo Cruz. 2017;112(4):237-246.
